# Internet-delivered interventions for personality disorders – A scoping review

**DOI:** 10.1016/j.invent.2022.100525

**Published:** 2022-04-01

**Authors:** Bram van der Boom, Nikolaos Boumparis, Tara Donker, Derek de Beurs, Arnoud Arntz, Heleen Riper

**Affiliations:** a Clinical Psychology Section, Department of Clinical, Neuro- and Developmental Psychology, Faculty of Behavioural and Movement Sciences, Vrije Universiteit, De Boelelaan 1105, 1081 HV Amsterdam, Netherlands; b Amsterdam Public Health Research Institute, PO Box 7057, 1007 MB Amsterdam, Netherlands; c Laboratory of Biological and Personality Psychology, Department of Psychology, University of Freiburg, Engelbergerstr, 41, D-79085 Freiburg im Breisgau, Germany; d Trimbos Institute—Netherlands Institute of Mental Health and Addiction, PO Box 725, 3500 AS Utrecht, Netherlands; e Department of Clinical Psychology, University of Amsterdam, Postbus 15804, 1001 NH Amsterdam, Netherlands; f GGZ inGeest Specialized Mental Health Care, VU University Medical Centre, De Boelelaan 1118, 1081 HZ Amsterdam, Netherlands; g Research Unit for Telepsychiatry and E-mental Health, Department of Clinical Research, University of Southern Denmark, Campusvej 55, DK-5230 Odense M, Denmark; h Faculty of Medicine, FI-20014, University of Turku, Turku, Finland

**Keywords:** Internet intervention, Personality disorder, Online, eHealth, Psychotherapy, Scoping review

## Abstract

**Background:**

Personality disorders (PDs) form a substantial part of the mental health disease burden. Effective therapies to treat PDs exist, but they are time-consuming, costly, and difficult to scale up. Delivery through the internet could facilitate the scalability of effective treatment methods.

**Objective:**

This review summarizes existing evidence on internet-delivered psychotherapy for personality disorders.

**Methods:**

Because few randomized controlled trials (RCTs) have been carried out, we conducted a scoping review. We performed a systematic literature search in PubMed, Embase, MEDLINE, CINAHL, PsycInfo, and Cochrane. Studies were selected if they conveyed research findings on internet-delivered PD interventions.

**Results:**

Eleven studies were included. The majority (*n* = 8) focused specifically on borderline personality disorder (BPD) and the other three on PD in general. The most frequently used form of intervention (*n* = 7) was the addition of a mobile app to a conventional evidence-based face-to-face treatment such as dialectical behavioral therapy (DBT). Most interventions (*n* = 8) were still in the development and piloting phase; only two RCTs were found. Usability and patient satisfaction were moderate to high in all studies. Three studies demonstrated significant decreases in borderline personality disorder symptoms.

The majority of the studies found were pilot or feasibility studies, most involving mobile apps offered in addition to face-to-face treatment. The add-ons were rated feasible, acceptable, and useful by patients. Reported challenges involved technical difficulties such as programming errors and bugs. Only 45% of the included studies reported on changes in PD symptoms, all showing reduction of symptoms and absence of adverse effects.

**Conclusions:**

This scoping review found that internet interventions for PD are still under-researched, although initial outcomes show promise. The outcomes also encourage future research in terms of developing internet interventions as an add-on to existing treatments, as well as working toward the creation and testing of more encompassing internet-delivered treatments for PD.

## Highlights

• Scoping review: internet interventions for personality disorders show promise.
• Online psychotherapy makes therapy more accessible for multiple mental health problems.
• Internet interventions could be of great use in treating personality disorders.
• Lack of RCTs with large sample sizes in research on eHealth for personality disorders.
• Research on internet interventions as an add-on to existing treatments is encouraged.

## 1 Introduction

Personality disorders (PDs) are characterized by an enduring and inflexible maladaptive pattern of behavior, cognition, impulses, and affect, leading to significant distress or impairment in daily life (Samuels, 2011) and are often associated with low quality of life, relatively high suicide rates, and high comorbidity with addiction (Feenstra et al., 2012; Verheul et al., 1998). PDs represent a heavy economic burden due to high demand on psychiatric, health, and social care services, high absenteeism, and productivity loss (Soeteman et al., 2008). They develop in late adolescence and often persist until late in life (Oltmanns and Balsis, 2011), thus forming a major public health problem, affecting around 10% of the adult general population (Samuels, 2011).

Although PDs were long considered untreatable due to their persistent and chronic nature, since the 1990s, several evidence-based psychotherapies have been introduced for treatment. Most notable are dialectical behavioral therapy (DBT) (Linehan et al., 2006), mentalization-based treatment (MBT) (Bateman and Fonagy, 2010), and schema therapy (ST) (Young et al., 2003). It is important to note that DBT and MBT were developed specifically to treat borderline personality disorder (BPD), whereas ST was developed for the full range of PDs.

Dialectical behavioral therapy is based on cognitive-behavioral therapy and was developed by Marsha Linehan in the late 1980s, when she found that CBT was not effective for BPD treatment (Linehan et al., 2006). DBT is based on the dialectical premise that everything is composed of opposites and that change occurs when there is a “dialogue” between opposing forces. DBT therapists work to resolve the contradiction between the need for change and the longing for self-acceptance, thus bringing about growth in the patient.

Mentalization-based therapy is an integrative form of psychotherapy, bringing together aspects of psychodynamic, cognitive-behavioral, systemic, and ecological approaches (Bateman and Fonagy, 2010). It is centered around *mentalization*, the ability to reflect on mental processes. Patients with BPD often lack this ability in various situations. Therapists help patients become aware of what is going on both in their minds and in the minds of others, leading to growth in understanding and more adaptive behavior.

Schema therapy combines techniques and theory from existing therapies, including psychoanalytic object-relations theory, attachment theory, cognitive-behavioral therapy and Gestalt therapy, into an integrative psychotherapy (Young et al., 2003). It is based on four main concepts: *basic emotional needs, early maladaptive schemas, coping styles,* and *schema modes*. The schemas, modes, and coping styles that cause problems in patients with PDs stem from unmet emotional needs. Techniques focus on childhood, present life and the therapeutic relationship; they may be experiential, cognitive, or behavioral. The therapeutic relationship is characterized by *limited reparenting*, which involves a supportive attitude, combined with empathic confrontation geared toward satisfying patients' unmet needs and healing maladaptive schemas, modes, and coping styles.

Even though these therapies differ in terms of their fundamental theories on mechanisms of therapeutic action, they share a clear theory explaining the feelings and behaviors of the patient. They help patients gain insight into what they can and cannot change, and provide opportunities to effect change. The general attitude and role of the therapists in these therapies are partly similar; an involved and active approach and the therapeutic relationship are central to each therapy (Oud et al., 2018).

Research has demonstrated that these treatments, applied individually or in group settings, effectively reduce PD symptoms in patients with BPD. A recent meta-analysis found that specialized treatments of BPD (DBT, MBT, ST, and transference-focused therapy) reduced BPD symptoms more effectively than treatment as usual, showing a medium effect size (Cohen's *d* = 0.59) (Oud et al., 2018). Thus far, however, very few studies have investigated the effects of such specialized treatments on patients with other types of PDs, although studies are currently being conducted into ST for such conditions. When it comes to cost-effectiveness, MBT has been little studied, and thus far with inconclusive results (Brazier et al., 2006); ST and DBT have been found cost-effective compared with other forms of therapy (Wetzelaer et al., 2016).

Although such psychotherapies are evidence-based, several problems hamper their widespread implementation. For example, due to the limited availability of trained therapists, predominantly living in to urbanized areas, only a limited number of patients have access (Samuels, 2011). Another challenge is that the therapies are time-consuming in terms of duration (routinely lasting one to three years) and intensity (commonly day-clinic and inpatient treatment) (Oud et al., 2018; Jacob and Arntz, 2013; Taylor et al., 2017; Perry et al., 1999; Biskin, 2015).

To overcome some of these barriers, clinicians have tested whether these specialized face-to-face psychotherapies would also prove effective if administered less intensively or in shorter forms. Although a limited number of such studies have been conducted, results have been promising (Nadort et al., 2009). An additional, more novel approach that can potentially overcome those barriers consists of internet-delivered treatment, which has already proven successful for several common mental health disorders, such as depression (Karyotaki et al., 2021), anxiety disorders (Andersson et al., 2019), and addiction (Riper et al., 2018; Ferreri et al., 2018). Internet interventions, which apply information technology in mental and behavioral health, vary widely in method of delivery. Methods include mobile applications, chat groups, video conferencing with a therapist, internet-delivered interventions with guided email contact, and unguided internet-delivered interventions (Ebert et al., 2018). Internet interventions can even serve as replacements to face-to-face (F2F) therapy, or as add-ons to such therapies, thus intensifying the dose (Cuijpers and Riper, 2020).

Internet-delivered interventions have several advantages over traditional therapy, including increased scalability and accessibility, in that many applications are not limited by time (they can be administered asynchronously) or space (they can be administered remotely) (Basnet et al., 2014). Despite the demonstrated success and value of internet-delivered interventions for many mental health disorders, there has been little development of such interventions for PDs, and only a limited number of studies have been conducted on their effectiveness. One possible reason is that PD treatments differ from those for common mental health disorders like depression and anxiety in that they are more complex and require more interaction between patient and therapist. Partly because the therapist–patient relationship is a key ingredient in the treatment (Kramer et al., 2020), the proven therapies may be more difficult to translate into suitable internet interventions. To provide more clarity, we decided to conduct a scoping review to investigate and structure the available evidence in this area of research. Our three specific objectives were (Samuels, 2011) to create an overview of the current state of the art in internet-delivered interventions for PDs, (Feenstra et al., 2012) to examine reported challenges and limitations of internet-delivered interventions for PDs, and (Verheul et al., 1998) to propose recommendations for future research in the field of eHealth and PD.

## 2 Methods

### 2.1 Overview

The scoping review consists of a systematic search of published, peer-reviewed literature for internet-delivered interventions for PDs, using the framework proposed by Arksey and O'Malley, and adhering to the recommended reporting guidelines (PRISMA) for scoping reviews as recently published by McGowan et al. (Arksey et al., 2005; Straus et al., 2020). The review is structured through five main processes: (Samuels, 2011) identifying the research question, (Feenstra et al., 2012) identifying relevant papers, (Verheul et al., 1998) selecting studies, (Soeteman et al., 2008) extracting data, and (Oltmanns and Balsis, 2011) collating and summarizing the data and results.

### 2.2 Inclusion and exclusion criteria

Five broad inclusion criteria were applied including studies targeting patients with a clinical diagnosis of any type of PD, assessed either by a clinician or by formal research assessments such as the M.I.N.I. diagnostic interview (Sheehan et al., 1998). Studies were further to include a digitally delivered treatment method, such as video conferencing, chat, emailing with a therapist, a mobile application, or internet-based interventions. Such digital interventions were to be either a self-contained protocolized treatment or an add-on to an analogue protocolized treatment. The studies were to report either on practical outcomes such as acceptability or feasibility of the digital intervention or on clinical outcomes such as symptom reduction, either by self-report or by clinician assessment. Studies with a variety of designs were eligible, ranging from single-arm pretest–posttest designs to RCT designs, and with quantitative as well as qualitative approaches. No language restrictions were applied.

For the purpose of this scoping review, we excluded descriptive studies without empirical data as well as studies focusing solely on vocal telephone interventions. Studies that were strictly observational or were focused on validating an online measurement tool, such as Tsanas et al. (Tsanas et al., 2016), were also excluded.

### 2.3 Search strategy

The search strategy terms included “personality disorder” and “internet intervention” (MESH terms). The following databases were searched for eligible studies published prior to August 2020: MEDLINE, PubMed, Embase, PsycInfo, CINAHL, and Cochrane Central Register of Controlled Trials (CENTRAL). The PubMed search string is provided in the Appendix A. After an initial search and the selection of potentially eligible studies, we manually screened applying reference lists to identify further titles.

### 2.4 Study selection

Titles and abstracts were screened independently by two reviewers (BvdB, NB) to identify papers that potentially met the inclusion criteria. Relevant studies chosen after title and abstract screening were reviewed in full text by the same two reviewers to assess inclusion eligibility. Any disputes were solved by discussion with the senior co-author (HR).

### 2.5 Data extraction

After inclusion of eligible studies, we extracted relevant data using a data extraction form (Table 1). All records were screened by two reviewers (BvdB, NB) to ensure accuracy. Disputes were resolved by discussion with the senior co-author (HR).

**Table 1:** Characteristics of included studies of internet-delivered interventions for personality disorders.

Study Year Country	Target disorder	Intervention	Sample size	Recruitment	Modality (stand-alone or blended)	Study design (control group)	Duration of intervention in number of sessions	Primary outcome (assessment measures)	Main findings
Rizvi et al., 2011 USA	BPD	DBT	*N* = 22	Outpatient	App (blended)	Pilot study (no control)	10–14 days of daily use of app	App usage (monitored by the app)	- App usage was high (participation rate 85%, SD = 0.14). - High acceptability and moderate-to-high patient satisfaction with the app - Reduction in emotional intensity from M = 6.83 (SD = 2.09) to M = 5.69 (SD = 2.31) - Decreased urge for substance use from M = 4.84 (SD = 3.23) to M = 3.95 (SD = 2.80) - Decrease in overall depression symptoms from 25.91 (SD = 9.90) to 20.32 (SD = 10.51), *d* = 0.55
Cristol, 2018 USA	BPD and depression	DBT	*N* = 1	Outpatient	App (blended)	Case report (no control)	3-month daily use of app	Patient satisfaction + treatment adherence (qualitative data)	- Patient satisfaction was reported high. - Engagement with the app and with treatment was high (*N* = 1).
Derks et al., 2019 NL	BPD	Biofeedback	*N* = 5	Inpatient	App (blended)	Feasibility study (no control)	During app development two days use of continuous data collection. These were then discussed F2F.	Usability (SUS)	- SUS rating was “good” for patients (mean score of 78.8, range 42.5–85), and “ok” for therapists (average score of 59.4, (range 30–85).
Helweg-Joergensen et al., 2019 DK	BPD	DBT	*N* = 16	Outpatient	App (blended)	Feasibility study (no control)	Ten months of individual or group DBT with add-on enabling daily use of app	Usability (SUS)	- High SUS ratings from patients (M = 81.2, SD = 9.9), and moderate SUS ratings from therapists (M = 68.3, SD = 14.3)
Austin et al., 2020 DK	BPD	DBT	*N* = 24	Outpatient	App (blended)	Pilot study (no control)	Twelve months of weekly group chat and available app use	Usage of the app, perceived helpfulness of DBT treatment, usefulness of the app, usefulness of the app in building therapist alliance (self-designed questionnaire using a 1–10 Likert scale)	- Usage of the app was high (20.3 weeks, SD = 6.3). - Treatment was perceived helpful (7.4 out of 10, SD = 6.3). - App was found useful (7.2 out of 10, SD = 2.2). - App was found useful in building alliance with the therapist (7.0 out of 10, SD = 2.3). - App was used moderately between sessions (6.9 out of 10, SD = 2.4).
Rhein, 2001 USA	PD and depression	Brief supportive therapy	*N* = 90	Outpatient	VC (stand-alone)	RCT (control: F2F therapy)	Eight 30-min sessions over a six-month period (VC)	Symptom change (HDS, BDI, SSAS), number of missed sessions, patient satisfaction	- Participants with PD receiving either VC or F2F treatment showed no significant difference in mean change of pre- and posttreatment scores for depression, anxiety, and global assessment of functioning. - Groups did not differ significantly in number of missed sessions or patient satisfaction. - Effect sizes were not provided.
Fassbinder et al., 2015 DE	BPD	ST	*N* = 1	Not specified	Website (blended)	Case report (no control)	Twelve months of weekly individual F2F therapy + add-on with 1 h recommended use of website	Symptom change (BPDSI, WHODAS, SMI)	- Significant improvement of symptoms on BPDSI, WHODAS 2.0 and SMI (*N* = 1)
Bilić et al., 2020 DE	PD and/or trauma disorders	DBT	*N* = 31	Inpatient	Website (stand-alone, but as follow-up to clinical treatment)	Pilot study (no control)	Three months, 12 weekly group chat sessions + on-demand individual chat sessions + use of exercises on website	Patient satisfaction (ZUF-8)	- Intervention was well-accepted, with 89.5% very or mostly satisfied. - Aim of the intervention, prevention of readmittance, was not compared with a control group.
Jacob et al., 2018 DK	BPD	ST	*N* = 14	Outpatient	Website (blended)	Feasibility study pre-post design (no control)	Twelve months of weekly individual F2F therapy + add-on with 1 h recommended use of website	BPD symptom reduction (BPDSI-IV), usage of the website (days per year)	- Usage was high (all patients used the program with an average of 80.7 days/year, SD = 72, range 12–288). - Over a period of one year, BPDSI scores were reduced by 9.6 points (SD = 9.7, *d* = 1) and BPD-CL scores by 29.9 (SD = 25.6, *d* = 1.2).
Zanarini et al., 2018 USA	BPD	Psycho-education	*N* = 80	Internet-based advertising /community	Website (stand-alone)	RCT (control: no intervention)	Twelve weeks' provision of online-accessible psychoeducation	BPD symptom reduction (ZRS-BPD), symptom severity (BEST)	- After 12 weeks, participants in treatment group reported a greater decline in impulsivity (*z* = −1.98; *p* = .048, *d =* 0.37) than control group. - At 12 months, they reported a greater decrease in cognitive symptoms (*z* = −3.20; *p* = .001, *d* = 0.46), affective symptoms (*z* = −2.31; *p* = .021, *d* = 0.69), impulsivity (*z* = −2.44; *p* = .015, *d* = 0.18), interpersonal difficulties (*z* = −2.15; *p* = .032, *d* = 0.22), and overall BPD symptoms (*z* = −2.11; *p* = .035, *d* = 0.42), as compared to control group. - At 12 months, the treatment group showed a significant decrease in symptom severity compared to baseline (*z* = −2.67; *p* = .008, 95% CI −8.70, −1.34, *d* = 0.28; secondary outcome)
Löhr et al., 2007 NO	PD, Social phobia, AD, Anxiety	CBT	*N* = 4	Outpatient	Email (blended)	Case report pre-post design (no control)	Nine months of weekly F2F sessions + weekly email add-on	User experience (qualitative data)	- Patients reported benefit from email as addition to usual treatment. They reported venting feelings and structuring therapy more easily with the email add-on.

Abbreviations: App, mobile phone application; AD, atypical depression; BPD, borderline personality disorder; CBT, cognitive-behavioral therapy; CI, confidence interval; DBT, dialectical behavior therapy; F2F, face-to-face; M, mean; *p*, *p*-value; PD, personality disorder; RCT, randomized controlled trial; SD, standard deviation; ST, schema therapy; VC, video conferencing; *z*, standard score

Assessment tools: BDI, Beck Depression Inventory; BEST, Borderline Evaluation of Severity over Time; BPD-CL, Borderline Personality Disorder Checklist; BPDSI, Borderline Personality Disorder Severity Index; BPDSI-IV, Borderline Personality Disorder Severity Index 4th edition; HDS, Hamilton Depression Scale; SMI, Schema Mode Inventory; SSAS, Spielberger State Anxiety Scales; WHODAS, World Health Organization Disability Assessment Schedule; ZRS-BPD, Zanarini Rating Scale for Borderline Personality Disorder; ZUF-8, Client Satisfaction Questionnaire - 8 items (German version).

### 2.6 Data analysis methods

We explored the current literature on data analysis methods and identified key studies. We then categorized our included studies based on sample, study design, type of intervention, and primary outcomes (see Table 1). We highlighted relevant results to devise suggestions regarding future research on internet interventions for patients with PDs.

## 3 Results

### 3.1 Study selection

**Fig. 1:**
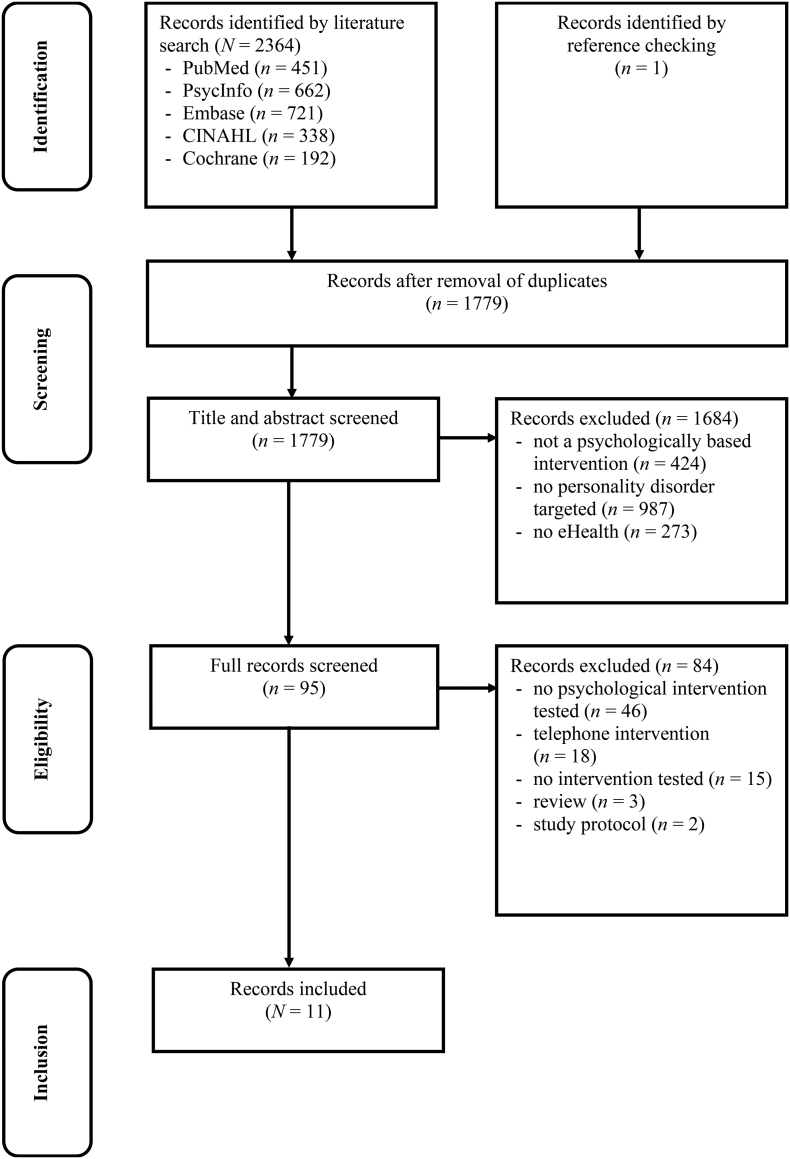
PRISMA flowchart of the study selection process.

The search resulted in 2364 titles (1778 after duplicate removal). We excluded 1193 based on title inspection. A further screening for potentially relevant studies in the reference lists of the identified studies resulted in one additional article (Rizvi et al., 2011). Of the remaining 586 records, 491 were excluded after abstract inspection. Of the 95 articles that were examined in full text, 84 were excluded. This left us with 11 studies for inclusion. The flowchart of the inclusion process following PRISMA guidelines is presented in Fig. 1.

### 3.2 Study characteristics

#### 3.2.1 General

Eleven eligibility criteria were included in the scoping review. Eight of them focused on patients with borderline personality disorder (BPD, with or without comorbid diagnoses), while three studies focused more broadly on personality disorders (with comorbid diagnoses), without differentiating between the patients' specific PD type. The studies were published between 2001 and 2020. See Table 1 for an overview of the characteristics of the included studies.

### 3.3 Targeted disorder

We found three studies (Rhein, 2001; Bilić et al., 2020; Löhr et al., 2007) that targeted any diagnosed PD, and eight studies specifically targeting BPD.

#### 3.3.1 Personality disorders in general

The three studies targeting an undifferentiated PD consisted of one case study (Löhr et al., 2007), one pilot study (Bilić et al., 2020), and one RCT (Rhein, 2001). Respectively they reported the testing of email as an add-on, a website program, and video conferencing.

#### 3.3.2 PD case study

The case study (Löhr et al., 2007) investigated the use of email as an add-on to a protocolized CBT treatment in a single-arm, pre-post study design, including four patients diagnosed with PDs as well as depression and anxiety disorders. All four patients received CBT F2F in varying frequency and amount, and received email communication as an adjunct treatment modality. The use of the email was a free choice; patients could use it just for organizational purposes, but were also free to discuss therapy-related issues. The agreement was that emails sent by the patient would be responded to promptly by the therapist. The study describes the experience of the four patients in their treatment setting. Results showed that email was seen as an effective and welcome adjunct to normal F2F treatment, helping patients to express themselves more easily, reducing formality in the therapeutic relationship, and increasing transparency. Challenges reported in this study were worries by the therapists about liability and confidentiality or privacy. The authors' opinion was that email therapy is unsuited for patients with more serious issues, such as suicidal ideations or psychotic symptoms.

#### 3.3.3 PD pilot study

Bilić et al. (2020) tested an internet-delivered program called Stay in Touch (SIT) in a pilot study. It was designed to prevent readmission of discharged inpatients (*N* = 31) with a PD. It included a weekly therapist-led chat group, individual chat sessions on demand, a crisis kit, psychoeducational material, exercises such as mindfulness, and additional DBT-based therapeutic modules. The pilot study found high rates of acceptance and patient satisfaction (89.5% were very or mostly satisfied). The therapist-led chat group was the module most intensely used by patients. Some 20% of participants were reportedly readmitted to a clinic within three months after discharge, but that rate was not compared to commonly known readmittance figures for patients with PD. Challenges involved browser- or device-based technical issues that could be resolved with minimal effort.

#### 3.3.4 PD randomized controlled trial

The RCT targeting PD tested video conferencing (VC) as a treatment modality. Rhein (2001) investigated the relationship between a PD diagnosis, treatment modality (live versus remote treatment), and mental health outcomes of veterans with a depressive disorder. In this study patients were randomized to either a F2F (*n* = 45) or a VC (n = 45) treatment of depression and aimed to see if patients with PD (*n* = 23) responded differently to patients without PD (*n* = 59). The study could not be found in any online preregistration databases. The primary outcome was treatment efficacy (with measures of depression, anxiety, and overall functioning). Relevant for the present review, the study showed no significant difference in treatment effect between the patients with or without PD, nor did it show any significant difference in treatment effect between the patients seen F2F and those seen through VC. The secondary outcomes were patient compliance and patient satisfaction. Results showed no significant difference in patient compliance between the F2F and VC modalities. However, for patients both with and without PD a trend was detected whereby remote treatment was linked to better compliance outcomes and to higher satisfaction in patients and psychiatrists alike as compared with face-to-face treatment. No challenges with respect to VC were reported.

### 3.4 Borderline personality disorder

Eight of our analyzed studies targeted borderline personality disorders specifically. Treatment designs included two case studies, one randomized controlled trial, two feasibility studies, and three pilot studies. The latter two types have a subtle difference. Feasibility studies examine whether internet-based interventions can be performed, whether they should be proceeded with, and how that can be done. Pilot studies may be considered a subset of feasibility studies. They focus on similar issues but have a specific design feature whereby a potential future study (or part of it) is conducted on a smaller scale, usually to test whether the components of the main study (such as recruitment, randomization, treatment, and follow-up) can all work well together (Eldridge et al., 2016). Thus, a pilot study is often a miniature version of a main study.

The modality used in the eight BPD studies was either a website program (Fassbinder et al., 2015; Jacob et al., 2018; Zanarini et al., 2018) or a mobile phone app (Rizvi et al., 2011; Cristol, 2018; Derks et al., 2019; Helweg-Joergensen et al., 2019; Austin et al., 2020). One study included depression as a comorbid diagnosis (Cristol, 2018) and one included substance use disorders (Rizvi et al., 2011). Only one study investigated an internet-delivered treatment modality as a stand-alone intervention (Zanarini et al., 2018); all others used their respective internet-delivered modality as an add-on or follow-up to an existing F2F treatment.

#### 3.4.1 BPD case studies

Cristol (2018) published a case report on one patient with BPD and major depressive disorder. As part of a DBT treatment, the patient searched for a suitable app to aid him in tracking his mood. The patient reported great help from the “over-the-counter” app Daylio, specifically due to not feeling the stigma of having to carry around a notebook and being identified as a mental health patient. The app also aided the therapy by providing more specific and current data on the triggers for the patient's intense mood change. No challenges were reported in this study.

In another case study, Fassbinder et al. (2015) introduced a web-based add-on program, Priovi, to an outpatient F2F schema therapeutic (ST) treatment for patients with BPD. The add-on program consisted of a website offering broad ST-based psychoeducation and therapeutic exercises. The study outlines the program and reports the experience of one patient with the program. That case report showed that the patient's BPD symptoms significantly improved over the course of the study and that patient satisfaction with the ad-on was high. No challenges or problems were reported.

#### 3.4.2 BPD feasibility studies

Jacob et al. (2018) tested the feasibility of the previously mentioned eHealth program, Priovi, as an add-on to F2F ST treatment for patients with BPD in a single-arm, non-randomized study (*N* = 14). Quantitative results showed that BPD symptoms improved significantly over time. Results from qualitative interviews showed that Priovi was positively received by patients and therapists alike. Challenges included technical difficulties that were easily fixed, usability problems such as patients disliking certain functions of the app, and patients experiencing some negative emotions with difficult topics and with bugs in the app.

Helweg-Joergensen et al. (2019) tested the feasibility of a mobile diary app as an add-on to F2F treatment among patients with BPD (*n* = 16) and their therapists (*n* = 23). The usability of the app, as measured by the Subjective Usability Scale (SUS), was found to be high (81.9) for patients, although therapists gave the app less favorable ratings (68.3). The authors also found that older age of users correlated with lower usability ratings. Reported challenges included minor technical difficulties, low levels of user technical skills in operating the app, and concerns among patients about the privacy of data collected by the app.

In their feasibility study, Derks et al. (2019) used a multicycle usability design to test a biofeedback app for patients with BPD (*N* = 5). The app gave biofeedback to help them improve their emotional awareness, something such patients often have difficulty with. The app scored well on usability (SUS average 78.8). The authors concluded that patients are enthusiastic and open to new mental health interventions delivered via mobile phones, enabling therapeutic benefits on a daily basis with little extra therapist effort. The app was intended as an add-on to conventional F2F therapy. Reported challenges included technical difficulties, which some patients experienced as stressful and demotivating, as well as usability issues, such as the app interface being experienced as suboptimal by some therapists.

#### 3.4.3 BPD pilot studies

Rizvi et al. (2011) conducted a pilot study (*N* = 22) examining the use of DBT Coach, an interactive app that facilitates skills coaching for patients with BPD and substance use disorders. The app was an add-on to a conventional F2F DBT therapy. The study demonstrated high acceptability and moderate-to-high patient satisfaction with the app. Clinically there was a significant reduction in emotional intensity (M = 6.83, SD = 2.09, to M = 5.69, SD = 2.31), a decreased urge for substance use (M = 4.84, SD = 3.23, to M = 3.95, SD = 2.80), and a decrease in overall depression symptoms (25.91, SD = 9.90, to 20.32, SD = 10.51). The only challenge reported by patients concerned technical errors.

Austin et al. (2020) conducted a pilot study investigating an app to enhance conventional F2F DBT for patients with PD. The app offered functions such as psychoeducation, CBT exercises and strategies, and self-monitoring. The results came from patient-completed questionnaires about their experience (*n* = 20), and a subgroup (*n* = 8) was also interviewed. Participants reported an overall positive experience with use of the app; it helped them access and implement DBT strategies and contributed to the therapeutic alliance. Quantitative responses from patients were congruent with those themes: 75% indicated that the app was very useful in DBT treatment and 80% reported that it helped create a good therapeutic alliance. Patients reported challenges including several technical issues, lack of IT support, and a lack of flexibility in certain app functions.

#### 3.4.4 BPD randomized controlled trial

The RCT conducted by Zanarini et al. (2018) tested the effect of a web-based psychoeducation program for young women with BPD. The study included 80 symptomatic female participants who met the DSM-IV criteria for BPD, 40 of whom were randomized to a treatment group that received internet-based psychoeducation and 40 to a waitlist control group. The study was preregistered in an online database. The primary outcome measure was BPD symptoms as assessed by the Zanarini Rating Scale for Borderline Personality Disorder (ZAN-BPD). The secondary outcome was borderline symptom severity, assessed by the Borderline Evaluation of Severity over Time (BEST). Regarding the primary outcome over the initial period of 12 weeks, the treatment group showed a greater decrease in one BPD symptom, impulsivity (*z* = −1.98; *p* = .048; 95% CI 0.23, 0.99), as compared with the control group. At 12 months, the treatment group showed better outcomes on all BPD symptoms, with greater decreases in cognitive symptoms (*z =* −3.20; *p* = .001; 95% CI 0.13, 0.62), affective symptoms (*z* = −2.31; *p* = .021; 95% CI 0.19, 0.87), impulsivity (*z* = −2.44; *p* = .015; 95% CI 0.19, 0.83), interpersonal difficulties (*z* = −2.15; *p* = .032; 95% CI 0.20, 0.93), and overall BPD symptoms (*z* = −2.11; *p* = .035; 95% CI -5.59, 0.20). Regarding the secondary outcome measure, the treatment group showed a decrease in symptom severity at 12 months compared to baseline, whereas the control group showed no difference; however, the difference in symptom severity between control and treatment groups was not significant. The article did not discuss any challenges encountered with the web-based psychoeducation program.

## 4 Discussion

### 4.1 Principal findings

The purpose of this review is to provide an overview of the current state of internet-delivered treatment for personality disorders (PDs), to report on any challenges in that treatment delivery, and to give guidance to future research. We included 11 studies in which all patients had PD as a primary diagnosis. Most studies (*n* = 8) focused specifically on the treatment for borderline personality disorder (BPD), which was to be expected, since BPD is one of the most common PDs (Hasin and Grant, 2015). Almost all studies (*n* = 9) used their internet intervention as an adjunct or follow-up to face-to-face (F2F) treatment and were based either on case reports or on small feasibility or pilot study designs. This is a logical first step in venturing into this field of research, and is similar to how digital interventions, such as internet-delivered or virtual reality interventions, have been developed for other mental disorders (North et al., 1997; Fletcher-Watson et al., 2016).

The relatively small number of studies displayed a wide variety in types of treatments (DBT, ST, CBT, psychoeducation), modalities (email, video conferencing, websites, apps), and populations (inpatient, outpatient, community). One of the two RCTs included here, which compared an internet intervention with an offline intervention, reported no significant difference in outcomes, symptoms, or patient satisfaction (Rhein, 2001). This may suggest that the internet-delivered intervention was comparable to the offline intervention with regard to the assessed outcomes, but the results must be interpreted with caution, given the small sample size.

The RCT that compared an internet-delivered intervention (unguided psychoeducation) with no intervention demonstrated a significantly better outcome for the internet-delivered intervention group, thus providing initial evidence that an internet-delivered intervention for PD could be more effective than no intervention (Zanarini et al., 2018).

These two findings, suggesting similar effects for internet-delivered in comparison with F2F interventions for PD, and larger effects for an internet-delivered intervention than for a no-intervention control condition, are in line with existing research on internet-delivered interventions in the context of other disorders, like depression and anxiety (Cuijpers and Riper, 2020; Batastini et al., 2021).

Although this is promising, the limited volume of studies shows that research on this topic is still in its infancy, and that much work needs to be done before we can make claims about the effectiveness of internet-delivered interventions for PD.

A similar conclusion was drawn in a recently published systematic review providing a meta analysis of the effect of smartphone applications targeting BPD symptoms (Ilagan et al., 2020). The review concluded that even though these interventions are user-friendly, they seem no more effective than treatment as usual and as such more research is needed.

The search results indicated that most studies have not gone beyond the piloting phase, and that over half assessed usability and feasibility as a primary outcome, while most also had relatively small sample sizes. Two studies also investigated the experiences of therapists with the digital intervention (Derks et al., 2019; Helweg-Joergensen et al., 2019). Therapist usability scores turned out lower than patient scores; Hellweg-Joergensen et al. (Helweg-Joergensen et al., 2019) ascribed this to mid-use changes made to the app, negatively affecting the therapists' SUS ratings, while Derks et al. (Cristol, 2018) also ascribed the therapists' lower ratings to bugs in the app. The identified difference in user experience between patients and therapists stresses the need to always incorporate therapists' user experience and feedback when researching internet interventions. Given that therapists' cooperation will be crucial for the eventual implementation of effective internet-delivered interventions, more efforts should be placed in informing them about the potential benefits associated with Internet interventions for those who do not wish or cannot engage with traditional healthcare systems (Watts et al., 2013).

Older users were generally more reluctant to use the app than younger ones, suggesting that when apps are implemented in care in general for PDs age might play an important factor. However, interestingly this is in contrast to what has been seen in depression research in which older age seems to be associated with increased intervention usage, increased duration spent in the intervention, and more positive perceptions of the interventions (Schneider et al., 2018).

Most of the internet-delivered interventions assessed here were employed in support of the existing evidence-based therapies DBT and ST. That is in line with the evidence base for those approaches as well as with their prolific use in the treatment for PD (Linehan et al., 2006; Oud et al., 2018). Why most studies have not investigated an encompassing internet-delivered treatment remains an open question. One reason might be that, in comparison with disorders like depression, the treatment for PD is more complex, and relies more on the personal interaction between patient and therapist. We found that most internet-delivered interventions applied common techniques like mood monitoring or psychoeducation that are also standard in the treatment for depression or anxiety disorders. Specific procedures that are considered important for the treatment for PD specifically, such as experiential techniques in ST (Young et al., 2006), have not yet been turned into unguided internet interventions for PD, with the exception of Priovi (Fassbinder et al., 2015; Jacob et al., 2018). Priovi integrates a number of unguided imagery techniques. We suggest that the overall lack of incorporation of imaginary techniques is due to the fact that they may be seen as too difficult, or too risky to translate into online modules. Our suggestion is based on the assumption that imaginary techniques can produce strong emotional reactions in patients triggering the urge of the therapist to be physically present in order to help channel such reactions as they occur. This assumption is confirmed by a recent qualitative study on Priovi (Köhne et al., 2020) in which patients report Priovi to be less emotional in interaction than psychotherapists leading to advantages as well as disadvantages. An advantage described was the ‘not feeling judged’ by Priovi. Feeling left alone with their emotions and feeling helpless to get out of these emotions without the support of a human being were labeled as disadvantages. Further exploration of transforming experiential techniques into internet-delivered interventions is in order. Experiential techniques provided through video conferencing were found to be useful and safe when applied in treating other disorders (Paulik et al., 2021). These findings combined suggest a feasible safe and effective online use of these techniques in the treatment of PD. In addition, we advocate the necessity of the incorporation of experiential techniques geared toward the creation of a more encompassing internet-delivered treatment for PD.

Specific procedures that are considered important for the treatment for PD specifically, such as experiential techniques in ST (Young et al., 2006), have not been turned into unguided internet interventions for PD, with the exception of Priovi (Fassbinder et al., 2015; Jacob et al., 2018). Priovi integrates a number of unguided imagery techniques. We think that the overall lack of incorporation of these techniques is due to the fact that they may be seen as too difficult, or too risky, to translate such techniques into online modules. The reason we suspect this is that such techniques can produce strong emotional reactions in patients, and therapists want to be present to help channel such reactions when they occur. This is also confirmed by a recent qualitative study of Priovi (Köhne et al., 2020). Patients report that Priovi is less emotional than a psychotherapist and that this has advantages and disadvantages. An advantage described was not feeling judged by Priovi. A reported disadvantage was feeling left alone with their emotions, feeling helpless to get out of these emotions without the support of a human being. Based on this we do advise further experimentation with transforming experiential techniques into internet-delivered interventions. We have also seen evidence that such experiential techniques, provided through video conferencing, were useful and safe when applied in treating other disorders (Paulik et al., 2021). These findings combined suggest a feasible safe and effective online use of these techniques in the treatment of PD. We also believe it is a necessary step in working toward the creation of a more encompassing internet-delivered treatment for PD.

Most studies (9 out of 11) discussed challenges that arose during the implementation of the various internet-delivered interventions. The majority of these were related to technical difficulties, such as programming errors and bugs, which sometimes caused frustration in patients and therapists alike (Rizvi et al., 2011; Derks et al., 2019; Helweg-Joergensen et al., 2019; Bilić et al., 2020; Jacob et al., 2018). Other challenges included usability issues (Derks et al., 2019; Jacob et al., 2018), privacy issues regarding the collection of data (Helweg-Joergensen et al., 2019), low satisfaction due to lower user technical skills (Helweg-Joergensen et al., 2019), and therapists' hampered ability to pick up on a patient's non-verbal communication in email exchanges (Löhr et al., 2007).

Except for this difficulty in reading non-verbal cues, none of the other challenges mentioned in these studies are insurmountable, and they can be effectively overcome in various ways. First, it is critical that the internet-based intervention modality in question should function well, in order to ensure more patient benefit and reduce frustration. That can be accomplished through quality programming and continuing IT support, though that would be costly. Second, to ensure high usability it is vital that feedback from patients and therapists be taken into account during the development of the internet-based intervention. Third, to ensure high satisfaction in patients and therapists with low technical skills, the use of the app must be made as easy and intuitive as possible, with improvements based on feedback from users. Finally, to alleviate patients' worries about privacy, it is essential that no more data than absolutely necessary be collected in implementing interventions, that all data collection meet high standards of privacy protection, and that patients be made aware of what data is collected and how their privacy is protected. Despite the challenges reported in the analyzed studies, patients and therapists still reported benefits and satisfaction from using the various internet-based interventions. Both these positive aspects should only improve further when the identified challenges are resolved.

### 4.2 Gaps and challenges

Our scoping review shows that there is little evidence available when it comes to using the internet to treat patients with personality disorders. The few studies that have been conducted report initial positive effects and satisfaction for patients. However, the lack of RCTs with large sample sizes confirms that further testing and application of internet interventions for PD is needed before any conclusions can be drawn.

Novel interventions should be developed and piloted among a sufficiently large group of clinicians and patients before large-scale studies are started. A further question is what the internet-delivered add-on components should ideally consist of: psychoeducation on issues such as mood, or active components of specialized BPD treatment techniques. We know of at least one intervention (Priovi) where pilots were followed by a large scale RCT comparing the effectiveness of care as usual for patients with BPD with and without Priovi add on (Klein et al., 2021). In this study the intervention group demonstrated no significant additional effect compared to treatment as usual (control group). This study should be followed by more RCT's. A step further would be to explore the possibility of offering a guided internet-delivered intervention without F2F contact. These kinds of interventions have been found to be helpful in the treatment of depression, but come with extra challenges in the context of PD, in view of the more difficult techniques and the greater focus on the therapist-patient relationship in PD. The possibility of replacing F2F with an entire treatment through video conferencing also seems feasible, as was seen in the study by Rhein (Rhein, 2001). We therefore also recommend that more large-scale RCTs be conducted to test VC treatment for PD.

### 4.3 Limitations

This review has a number of limitations. First, because few studies have been conducted, we could not perform a meta-analysis to produce a quantitative analytical synthesis of the evidence. However, we have followed the recommended reporting guidelines as recently published by PRISMA (Straus et al., 2020). Second, as yet, criteria to assess the quality of scoping reviews are not standardized, which is likely due to the relative newness of this type of review (Sucharew and Macaluso, 2019). Third, the number of participants in many of the included studies was small whether they entailed proof of concept studies addressing a number of outcomes, or clinical trial designs. This probably resulted in underpowered studies, unable to detect subtle effects of the interventions studied. Working with small samples also reduces the precision and accuracy of statistical results, thus limiting our interpretation of those results (Leon et al., 2011). A fourth limitation is that no studies examined the long-term effects of internet-delivered interventions for PD, making it impossible for us to report on longer-term effects. Lastly, as the field is still in its infancy, some novel studies may have remained unpublished, making our review less complete than it might have been.

## 5 Conclusion

Our scoping review has provided insights into the developed and tested internet interventions for patients with personality disorders. Analysis of 11 studies found that internet interventions for PD are still under-researched, although initial outcomes show promise. The outcomes also encourage future research in terms of developing internet interventions as an add-on to existing treatments, as well as working toward the creation and testing of more encompassing internet-delivered treatments of PD.

## Declaration of competing interest

The authors declare that they have no known competing financial interests or personal relationships that could have appeared to influence the work reported in this paper.

## Appendix A Search String

Search String Image 1
