# Supplementary material for: Internet-delivered interventions for personality disorders – A scoping review
Source: Internet Interv. 2022 Apr 1;28:100525. doi: 10.1016/j.invent.2022.100525 (PMC9018158; doi:10.1016/j.invent.2022.100525)
Supplement: Supplementary file 1 — Search String [file mmc1.docx]

Appendix

The following search keywords were used : (Personality Disorders OR personality disorder* OR antiso*  OR borderline* OR compulsive OR histrion* OR paranoid* OR passive-aggressive* OR schizoid* OR schizotypal*  OR narciss* OR avoidant OR dependent AND disorder*)

AND

("Telemedicine"  OR "Telenursing"  OR "Public health informatics" OR "Therapy, Computer-Assisted" OR "Videoconferencing" OR econsult* OR e-consult* OR mobile health* OR mhealth* OR m- health*  OR telehealth* OR tele-health OR remote consult*  OR teleconsult* OR  tele-consult* OR telenursing  OR tele-nursing OR telediagnos* OR tele-diagnos* OR telemedic* OR “tele-medic*” OR telemonitor* OR “tele-monitor*” OR ehealth* OR “e-health*” OR telecare OR “tele-care” OR digital consult*” OR “online consult*” OR “internet consult*” OR “internet-based consult*” OR “web-based consult*” OR “mobile consult*” OR cyberconsult* OR etherap* OR “e-therap*” OR “mobile therap*” “web-based therap*” OR “distance therap*” OR cybertherap* OR videoteleconferenc* OR  “internet discussion group*” OR “internet- based discussion group*” OR “web-based support group*” OR “online support group*” OR “internet support group*” OR “internet-based support group*” OR “video group*” OR “video session*” OR “tele-session*” OR “internet session*” OR “internet group*” OR “internet-based group*” OR “online session*” OR “online group*” OR “web-based session*” OR “web- based group*” OR “e-coach*” OR skype OR interapy.
